# A Randomness Detection Method of ZigBee Protocol in a Wireless Sensor Network

**DOI:** 10.3390/s18113962

**Published:** 2018-11-15

**Authors:** Yongli Tang, Huanhuan Lian, Lixiang Li, Xiaojun Wang, Xixi Yan

**Affiliations:** 1 College of Computer Science and Technology, Henan Polytechnic University, Jiaozuo 454000, China; yltang@hpu.edu.cn (Y.T.); hhl9307@163.com (H.L.); 2 School of Cyberspace Security, Beijing University of Posts and Telecommunications, Beijing 100876, China; lixiang@bupt.edu.cn; 3 School of Electronic Engineering, Dublin City University, Dublin 9, Ireland; xiaojun.wang@dcu.ie

**Keywords:** wireless sensor network, ZigBee protocol, randomness detection, matrix probability test

## Abstract

This study investigates the randomness detection of cryptographic algorithms in network security. To effectively test and verify the security of ZigBee protocol in the Internet of Things, the study combines with the characteristics of ZigBee networks, and it rationally organizes and divides test modes based on the binary matrix rank theory test. Then this paper proposes a randomness detection method of ZigBee protocol in a wireless sensor network. The proposed method solves the one-sidedness that the binary matrix rank test simply assesses random sequences by linear correlation. The proposed assessment method can effectively appraise whether the ZigBee protocol has an encryption mechanism and encryption strength. Simulation results show that this method has the characteristics of fewer errors and high reliability.

## 1. Introduction

As an important part of the Internet of Things, ZigBee technology is widely used in industrial control, agriculture, environmental science, and other fields due to its low power, low cost, and low latency. As a result, the security of ZigBee technology has become a popular research topic. Currently, the security of the ZigBee protocol lacks an effective assessment method since the ZigBee protocol contains an optional security encryption mechanism, an encryption algorithm at the high-definition level, and many other encryption levels with diverse characteristics. From the perspective of computational complexity and cryptography, random numbers are the basis of the security of cryptographic algorithms, and there is no other more important issue than this, as the famous cryptographer Bruce Schneider said [1]. In cryptographic and authentication protocol algorithms, a random number sequence needs to be used as the key, and randomness assessment is important in studying the security of a cryptographic algorithm. Therefore, the study of randomness assessment in relation to ZigBee has important theoretical implications for ZigBee technology and the security of the Internet of Things.

Randomness is closely related to cryptographic security, and the security of a cryptographic algorithm can be evaluated according to the randomness of the algorithm. Randomness test of a sequence, in essence, is a test of whether it is truly random [2]. Schneider stated that a random sequence must be able to pass all the correct randomness tests, must be unpredictable, and must produce results that cannot be repeated.

At present, there are many randomness tests. Marsaglia proposed the Diehard Battery test set [3], but it has high requirements for sequence length, uses a small number of samples for reference distribution that are difficult to calculate, and is stricter in reference distribution. The NIST SP 800-22 specification [4] recommended 15 statistical tests for randomness testing. All the tests are used in the same manner to compare $P_{\text{-}value}$ value and use the significance level $p_{c}$ to determine whether a sequence is random, but they do not deal systematically with the relationship between a statistical test and randomness or between the sample size and the conclusion’s credibility. The author(s) of Ref. [5] proposed that a run test cannot reflect the essential characteristics of randomness. In the literature [6,7,8], the parameter set of the poker test and the two elements deduction test were also reported. These provide optimization methods and ideas for a testing project with references, but they make it difficult to guide the operations and are unsuitable for the resource-constrained system. The author(s) of Ref. [9] used the reference distribution of a Fourier transformation test to approximate a binomial distribution; the threshold value and variance are optimized, but there are still some errors. The author(s) of Ref. [10] showed that the frequency and the non-superposition pattern matching tests have a strong hardware implementation, but the length of the sequence is higher and the testing time is longer. The binary matrix rank test and poker test involve complex mathematical transformations, they incur hardware implementation resource overhead, the test speed is slow, and they cannot meet real-time requirements. In the literature [11,12,13,14], a new randomness testing method under the condition of known encryption algorithm and structure used in the assessed network was proposed, but it cannot be applied to networks where the encryption algorithm is unknown or self-defined. Literatures [15,16] gave some practical systems which proposed a practical development of a communication infrastructure.

In the literature [17,18] it was shown that a serial test is better than using both the poker test and the sequence pair test effect, but the binary matrix rank test has higher eliminating ability than the serial test, eliminates the random sequence as soon as possible, and improves the efficiency. Other studies [10,19] showed that the binary matrix test, methods rank test, and Lempel-Ziv test are similar. Although the Lempel–Ziv test is believed to include a frequency test, runs test, compression test, or other spectrum inspection, and the test effect was significant, its applicability is limited because of an obvious shortcoming, that is the statistics of the distribution function cannot be accurately defined. However, when the construction matrix of the binary matrix is full rank, the $P_{\text{-}value}$ value is even lower than the full rank $P_{\text{-}value}$ value; the test can be judged only by whether the linear correlation chip is random, but it cannot judge the strength of the randomness. In addition, regarding the functional testing of ZigBee products and the improvement of the ZigBee network encryption algorithm, the security and validity of the research are difficult to verify in practical applications.

The author(s) of Ref. [20] illustrated a randomness testing method that was suitable for a system of no-encryption measures. It used frequency detection and frequency detection within blocks algorithms to analyze and detect the encryption effect of cryptographic algorithms. Frequency detection has the advantages of simple realization, distinguishing degrees, small sample requirement, and fast calculation. So it can quickly identify whether the ZigBee network adopts encrypted transmission. In the present paper, the study was based on Ref. [20] that used a frequency detection algorithm to conduct the frequency test. Differently, this study also used matrix probability detection to test the randomness strength of the sequence. The matrix probability test algorithm appraises the randomness of the original sequence by examining the probability of a randomly distributed generation matrix, which reduces the number of complex mathematical calculations and uses few hardware resources. In addition, it can detect a significantly nonrandom sequence, which gives test results with a lower error rate. This method can effectively appraise not only whether the ZigBee network is encrypted but also the strength of encryption.

In summary, to satisfy the ZigBee protocol randomness test with high efficiency and real-time requirements, this study combines the characteristics of the ZigBee protocol, from the viewpoint of reducing hardware implementation cost and error, to improve the binary matrix rank test algorithm. This paper proposes a randomness test method for the ZigBee protocol based on the matrix probability test. This can determine effectively for a ZigBee network whether encryption is effective as well as the strength of the encryption, to achieve a ZigBee protocol data transmission security test.

The remainder of this paper is as follows. Section 2 presents ZigBee technology. Section 3 proposes a sensible test method and a randomness test algorithm based on the matrix probability test. Section 4 verifies the reliability and rationality of the algorithm through simulation experiments. Finally, Section 5 presents the main conclusions.

## 2. ZigBee Technology

ZigBee is a new wireless communication technology with small size, low power consumption, low cost, and low transmission rate. It is one of the most widely used transport protocols in Internet of Things (IOT) perception layer devices.

### 2.1. ZigBee Technical Characteristics

ZigBee technology is developed based on IEEE802.15.4. The underlying technology of ZigBee is based on IEEE802.15.4, which means that the physical layer (PHY) and medium access control (MAC) layer protocols are defined by IEEE802.15.4, so ZigBee Protocol has the advantages of low power consumption, low cost, and low complexity. While the network layer, application layer, and security service layer were developed by the ZigBee Technology Alliance, the network layer was standardized. ZigBee has the following features:

(1) Low power consumption: This is a unique advantage of the ZigBee protocol. In the sleep state, only a few uw of power is consumed; in short-distance transmission, only tens of mw of power is consumed; in standby state, two dry batteries can work for 6 months or more;
(2) Low cost: The module cost of the ZigBee network is low, and the code is open source;
(3) Large network capacity: A ZigBee network can accommodate up to 65,535 sensors and can be expanded by a 64-bit IEEE address with a considerable capacity;
(4) Reliability: In order to reduce competition and conflicts, support collision avoidance when data is transmitted, and support full confirmation mode, each data packet needs to receive confirmation information, and if there is a problem, it supports retransmission.
(5) Security: ZigBee supports packet integrity check function, authentication, and authentication. It can also determine different security attributes in different scenarios according to needs;
(6) Flexible working frequency band: There are 16 spread spectrum communication channels, and there are three common frequency bands, namely 2.4 GHz, 868 MHz (Europe), and 915 MHz (USA).

### 2.2. ZigBee Protocol Security Mechanism

The security functions in the ZigBee network can be summarized as two aspects: (1) ensuring that legitimate users can use normally without being illegally destroyed; and (2) preventing illegal use and the theft of information. These two functions then require ZigBee security to provide these security services: key establishment, key transfer, frame protection, and device management. Each layer of the ZigBee protocol has its own security mechanism that combines the security functions of each layer to form the security service layer of the ZigBee protocol. The ZigBee network equipment is simple, low-cost, and self-organizing within the network, so complex security mechanisms cannot be used. The protocol layers need to trust each other between different applications of the same device, so the key materials between the protocol layers are shared, which saves a lot of key storage space.

The security mechanism of the ZigBee protocol has the following principles [21]: (1) the layer that originally generated the frame is responsible for the initial encryption; (2) all frames are secured by using the network (NWK) layer (except for routing and new devices); (3) the same device in different protocol layers can reuse the key; (4) end-to-end security is enabled so that only the source device and the target device can access their shared key; (5) to simplify the network, all devices in a network and all layers in a device should use the same level of security.

The security architecture of the ZigBee protocol includes two levels of security: NWK and application support-sub layer (APS) are responsible for the security of frames at their own layers. In addition, APS provides the establishment and maintenance of secure services and ZigBee device object (ZDO) manages device security policies and security configurations.

When the frame generated by the application layer (APL) needs to be encrypted, the APS sublayer handles the security of the frame. The security of APS layer frames is based on a connection key or a network key. The APS layer is also responsible for providing key establishment, key transport, and device management services for application objects and ZDO [21].

The network layer is responsible for securely transmitting output frames and receiving input frames securely. The upper layer controls the security operations of the NWK layer by establishing appropriate keys (active and alternate network keys) and frame counters, and establishes a security level. The frame security mechanism of the NWK layer uses the advanced encryption standard (AES) and the continuous conduction mode (CCM*) mechanism [21,22].

## 3. Randomness Tests Method of ZigBee Protocol Based on the Matrix Probability Test

### 3.1. Method of Dividing a Sequence and Testing Its Organization

Randomness testing of ZigBee using the matrix probability test is performed by examining the distribution of the probability judgment matrix. The probability distribution of the unpredictability of the measurement of a pseudorandom sequence is a very important index. In the actual testing process, the probability distribution reflects the unstable nature of existence. Therefore, to make the randomness test more efficient and reliable, this paper proposes a sensible test organization method that can investigate the stability of random sequences effectively.

The frequency test is the most basic method proposed by NIST SP800-22. It has the advantages of simple realization, distinguishing degrees, small sample requirement, and fast calculation. In order to identify whether the transmission is encrypted in the ZigBee network, the frequency test can be used for sequence in priori detection. If a sample does not pass the frequency test, it can be considered that the sequences are not random, obviating the need to perform other tests. If the sample passes the frequency test, the matrix probability test can be conducted for its randomness strength.

Meanwhile, National Institute of Standards and Technology (NIST) analysis of the dependence of the frequency of inspection and the test matrix concluded that the dependencies between them are very small. Previous studies [23,24] showed that the correlation between the frequency and matrix tests is *R* = 0.025 < 0.2, showing some dependency or relevance. Therefore, the method of combining the frequency and matrix tests not only will not affect the correlation of test results, but also will make the test more convincing and more efficient.

The specific tests are organized as follows:

1. First, the ZigBee sequence test samples were separated into groups and each sample was given a number, 1, 2, 3, …, *N*.
2. Each set of samples was tested with frequency tests and various parts of the sequence were inspected randomly. If a sample did not pass this test, the test ended; if the sample passed this test, we proceeded to the next step.
3. Cumulative test mode was used and the number of samples was increased successively (that is, each time an additional set of samples was included, up to the sample N group to include the entire sequence) to conduct the matrix probability test in order to measure the stability of a random sequence and verify the degree of randomness of the ZigBee encryption algorithm.

Note that this method is based on the premise of the frequency test. First test whether the measured series ratio is 0 or 1 and whether the code is 1/2. In the more egalitarian premise test sequence with a 0 code, using a test matrix probability distribution can investigate further whether the 0 or 1 sequence follows a uniform random distribution.

As the actual testing process uses a multi-sequence testing strategy, when evaluating the performance quality of random sequences, *P_-value_* value can be used to measure the uniformity of the value distribution method, as proposed by NIST SP800-22. The specific method is to select a number of sequences generated by the encryption algorithm for the randomness test and produce a value corresponding to the *P_-value_* set, evaluating the pass rate and distribution of cryptographic algorithms by examining the *P_-value_*. Because the *P_-value_* is a real number between zero and one, the interval (0, 1) can be divided into ten subintervals. When the number of sequences is sufficiently large, *P_-value_* must be evenly distributed into ten intervals. For the frequency statistic, $V_{i}$, for each interval, $V_{1}+V_{2}+\cdots V_{10}=m$ (*m* is the number of sequences), calculate the following:

(1) $$\chi^{2}=\sum_{i=1}^{10}\frac{{\left(v_{i}-m/10\right)}^{2}}{m/10}\;$$

(2) $$P_{\text{-}valueT}=igamc\left(\frac{9}{2},\frac{\chi^{2}}{2}\right)\;$$

If $P_{\text{-}valueT}>0.0001$, then the sequence is consistent with a uniform distribution, i.e., good randomness.

If a ZigBee sample cannot pass the frequency test, it can be considered that the randomness is poor, the ZigBee network encryption measures are not used, and the data is likely to be transmitted in plain text. If the sample passes the frequency test and follows a uniform distribution, this indicates that the ZigBee network has high randomness, and we can proceed to perform the matrix probability test.

If a sample does not pass the matrix probability test, it can be inferred that the ZigBee network encryption mechanism is either not properly implemented or not implemented; if it passes the matrix probability test, it demonstrates good randomness of the ZigBee network, and implies that the ZigBee network securely implemented encryption mechanisms.

### 3.2. Sample Testing for a Random Probability Algorithm Based on a Matrix

The linear correlation test is a very important indicator of a random sequence binary matrix in the rank test. In binary matrix rank inspection, using the number of the square full rank statistical test sequence structure $f_{m}$, the number of *m* − 1 rank matrices is determined to be $f_{m-1}$ by examining the matrix of rows and linear correlation columns to determine the randomness of the sequence. However, compared to traditional information systems, the ZigBee network has a higher packet loss rate, and the missing data packets and their order will have a greater impact on the rank of the matrix, hence the binary matrix rank test results may not be a true reflection of ZigBee random sequences. This test method is not fully applicable to the ZigBee network. According to NIST, if the square matrix rank test order is 32, then the square will show a lower linear correlation, and in some cases, the binary matrix rank test can yield a conclusion that the given sequence is nonrandom, hence it might not detect a significantly nonrandom sequence.

Therefore, referencing the binary matrix rank test and combining it with the characteristics of ZigBee, a test method based on the probability matrix test is proposed. This method is performed by testing whether a new generation matrix **C** in the proportion of zero is similar to the assumption that the sequence has a P_c_ probability random condition, thus judging random test sequences.

For each position (*i*, *j*), if the probability of the symbol 0 and 1 appearing in matrices **A** and **B** are $p_{a}$ and $p_{b}$, respectively, and the symbols 0 and 1 are randomly distributed in **A** and **B** in terms of position, then the probability of the symbol 0 appearing in **C** for each position is $P_{\text{-}valueT}$, where C = AB.

Proposition: The probability is $P_{\text{-}valueT}$, where m is square of order.

Proof: For generating square matrix **C**, one element is $c_{ij}=\sum_{k=1}^{m}a_{it}b_{tj}$.

The probability matrix **C** appears as

(3) $$\begin{array}{ll} P_{c} & =\left(c_{ij}=0\right)\\ & =\prod_{t=1}^{m}P\left(a_{it}=0\;or\;b_{tj}=0\right)\\ & =\prod_{t=1}^{m}\left(\begin{array}{l} P\left(a_{it}=1\right)P\left(b_{tj}=0\right)\\ +P\left(a_{it}=0\right)P\left(b_{tj}=1\right)\\ +P\left(a_{it}=0\right)P\left(b_{tj}=0\right) \end{array}\right)\;\\ & =\prod_{t=1}^{m}\left(\left(1-P_{a}\right)P_{b}+P_{a}\left(1-P_{b}\right)+P_{a}P_{b}\right)\\ & =\prod_{t=1}^{m}\left(P_{a}+P_{b}-P_{a}P_{b}\right)\\ & ={\left(P_{a}+P_{b}-P_{a}P_{b}\right)}^{m} \end{array}$$

Based on the matrix, the probability test algorithm is as follows:

1. With the output sequence of length n as the ZigBee test sequence, divide the test sequence sequentially into $M=\left\lfloor \frac{n}{2}\right\rfloor$ bit disjoint blocks and obtain two blocks, discarding the remaining bits. The remaining n−2×M bits are discarded.
2. The two **M** bit blocks, configured as two m×m matrices, are defined as the square matrices **A** and **B**, respectively, with two statistical proportions of a 0 phalanx, where in $M=m^{2}$.
  The proportion of the number 0 in matrices **A**, **B**:
  (4) $$P_{a}=P\left(a_{it}=0\right)=\frac{the\;number\;of\;0s\mathrm{in}\;the\;matrix\;A}{m^{2}}\;$$
  (5) $$P_{b}=P\left(b_{tj}=0\right)=\frac{the\;number\;of\;0s\mathrm{in}\;the\;matrix\;B}{m^{2}}\;$$
3. The matrices **A** and **B** when multiplied yield the newly generated matrix, C = AB.
  The proportion of the number 0 in matrix **C**: $P_{c}{}_{\left(\mathrm{obs}\right)}$:
  (6) $$P_{c}{}_{\left(\mathrm{obs}\right)}=P\left(a_{ij}=0\right)=\frac{the\;number\;of\;0s\mathrm{in}\;the\;matrix\;C}{m^{2}}\;$$
4. For the ratio of statistics $P_{a}$ and $P_{b}$, calculate the corresponding expectations $P_{c}={\left(P_{a}+P_{b}-P_{a}P_{b}\right)}^{m}$.

Theoretically, if the statistic $P_{c}{}_{\left(\mathrm{obs}\right)}$ and expectation $P_{c}$ are equal, then this sequence passed the inspection.

In fact, it is not possible for each sequence produced to be entirely consistent with the desired result. Thus, the concept of statistical confidence interval is introduced. When the interval $P_{c}{}_{\left(\mathrm{obs}\right)}\in \left(P_{c}-3\sqrt{\alpha \left(1-\alpha \right)/n},P_{c}+3\sqrt{\alpha \left(1-\alpha \right)/n}\right)$ (*n* is a sequence number) the sequence is considered to have passed the test; otherwise, it fails the test.

The matrix probability test algorithm, by examining the probability of a randomly distributed generation matrix to determine the randomness of the original sequence and reducing the number of complex mathematical calculations, uses few hardware resources. Then, the low overhead satisfies the ZigBee randomness testing process and meets real-time resource requirement limitations. When the magnitude of the deviation comparative statistical results meets the expectations, not only can it detect a nonrandom sequence effectively, but it can also detect a significantly nonrandom sequence, with lower error rate of test results, higher accuracy, and more suitability for ZigBee detection of random sequences.

## 4. Simulations and Analysis

### 4.1. Simulation Environment

In this study, using MATLAB simulation software, devices from three different manufacturers were selected to transfer data as samples using the ZigBee protocol. ZigBee devices from manufacturer A deployed no encryption measures, ZigBee devices from manufacturer B used a lightweight cryptographic algorithm for data encryption, and ZigBee device from manufacturer C used the AES CCM* encryption algorithm for data encryption.

Better statistical test sample parameters were used to ensure comparability of results. In the tests, the same node and location but different data for analysis were selected. The specific randomness testing parameters were as follows:

1. Sample size: 50 groups drawn from sample sequences of 50 bytes, with one byte extracted from each set of samples from the 50 ZigBee sequence payload data to comprise a 50-byteZigBee sequence.
2. Significance level: In accordance with NIST standards for testing, set the value of the significance level at 0.01.
3. Sample start, sample end: When taking samples, use the same location for analysis of experimental data payloads.

### 4.2. Simulation Analysis and Conclusions

(1) Algorithm correctness verification: 50 randomly selected sample data from each set of data are used in order to perform the matrix probability rank test, using the testing samples in Table 1, where n=50, α=0.01 and $P_{c}{}_{\left(\mathrm{obs}\right)}\in \left(P_{c}-\;0.0422,P_{c}+\;0.0422\right)$.

Through data analysis, the probability test when the difference between the $P^{\prime }$ matrix expectations and the statistical value is not within the desired range, results in a matrix of $P_{\text{-}value}$-rank test values of less than 0.01, indicating poor sequence randomness; when $P^{\prime }$ is in the interval, the matrix rank test yields $P_{\text{-}value}$ values greater than 0.01, indicating better sequence randomness. Experiments show that the stochastic conclusions are consistent with the matrix probability test being able to detect random sequences accurately.

**Figure 1:**
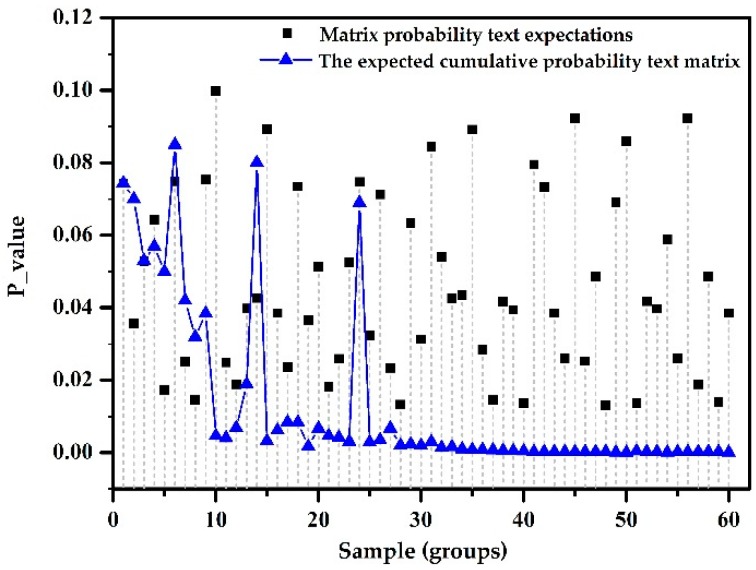
Organization superiority with reasonable verification.

(2) Testing organization superiority with reasonable verification: 60 randomly selected groups of sample sequences were used for a single test and successively in an accumulative manner, with the test results shown in Figure 1.

Experimental Analysis: When using a single test sample group, the value of $p_{c}$ was randomly distributed in (0, 0.1). With accumulative successive test sample set, it is evident that the number of samples from the stationary time series had volatility of 6, 14, 24, or larger, and with an increase in the sample group, the *P* value was gradually stabilized. Experiments show that during the randomness test, using the successive accumulation mode can be more intuitive for testing stationary random sequences; this method is more reasonable and advantageous.

**Figure 2:**
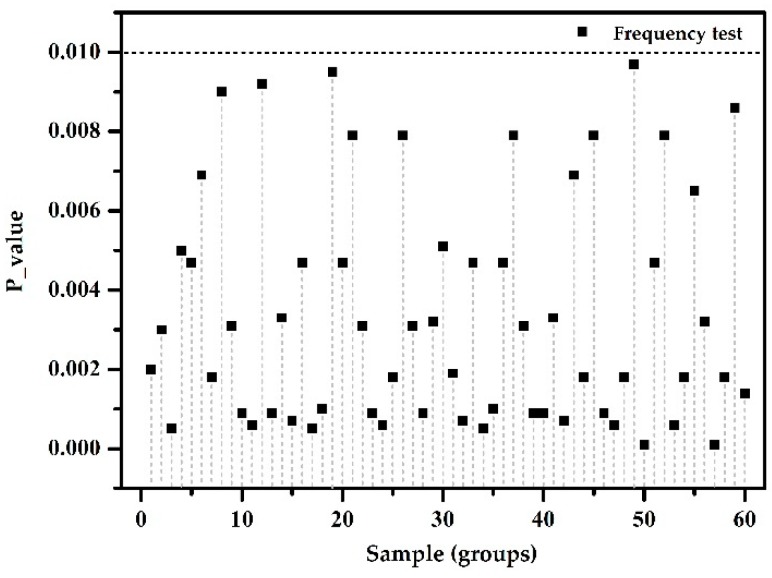
No encryption measures.

(3) Encryption measures are not used in the deployment of ZigBee devices, with randomness test results shown in Figure 2.

The experimental results: In this test, with a frequency of inspection of $P_{\text{-}value}<0.01$, the sample cannot pass the test. The test showed that this sample has poor randomness; hence the ZigBee data on the network are likely to be transmitted in plain text. The randomness detection method can successfully determine the ZigBee protocol for unencrypted data transmission.

**Figure 3:**
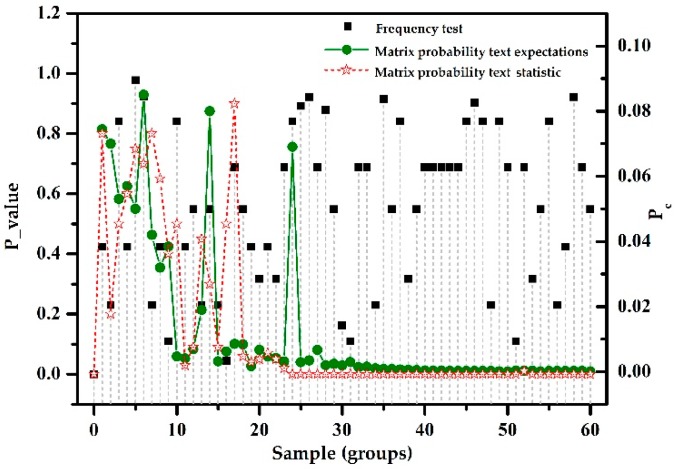
Lightweight encryption algorithm.

(4) ZigBee devices from manufacturer B used the modified Zug lightweight encryption algorithm for data encryption. Among these, $P_{\text{-}value}$ represents the result of the frequency test, and $p_{c}$ represents the result of the matrix probability test. The results of the randomness tests are shown in Figure 3.

The experimental analysis is as follows. In this test, when the frequency test $P_{\text{-}value}$ > 0.01, and was fairly evenly distributed in (0, 1), the statistical calculations yield $P_{\text{-}valueT}$ = 0.0187 > 0.0001. For the matrix probability test, the deviation of the $p_{c}$ statistical value and the expected value of the sample were relatively large, and the sample was not stable. Experiments showed that a test sample can pass the frequency test, but fail to pass the matrix probability test, hence it is considered that the sample is random, but the randomness stability and encryption strength are poor. The corresponding ZigBee protocol implemented the encryption mechanism, but because of the use of a lightweight encryption algorithm, there was a lack of encryption strength. Randomness test methods can determine whether the ZigBee protocol data transmission are encrypted or not and the strength of the encryption.

**Figure 4:**
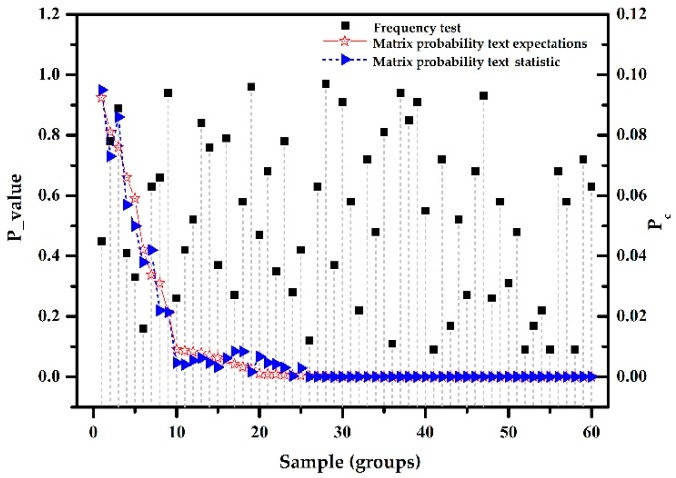
AES CCM* encryption algorithm.

(5) ZigBee devices from manufacturer C used the default AES CCM* encryption algorithm for data encryption. The results of the randomness tests are shown in Figure 4.

The experimental analysis is as follows. When the frequency test yielded $P_{\text{-}value}>0.01$ and was fairly evenly distributed in (0, 1), the statistical calculations yielded $P_{\text{-}valueT}$ = 0.0296 > 0.0001. For the matrix probability test, the statistical value of the sample, $p_{c}$, was basically consistent with the expected. Since the experiments showed that the test sample can pass the frequency and matrix probability tests, it is considered a good random sample because the randomness stability and encryption strength are high. The corresponding ZigBee network implemented the encryption mechanism. The security of the cipher is also very high because the AES CCM* encryption mode of the ZigBee protocol has better confidentiality. The randomness tests method can accurately determine whether the ZigBee protocol data transmission are encrypted or not and if encrypted, the strength of the encryption.

The above experimental results show that the algorithms that pass the matrix probability test or fail to pass have obvious differences in encryption strength, and the security is also different. Therefore, the detection method based on the matrix probability test can determine effectively for a ZigBee network whether encryption is effective as well as the strength of the encryption, to achieve a ZigBee protocol data transmission security test.

## 5. Conclusions

The randomness of the ZigBee protocol was studied from the viewpoint of the security of cryptographic algorithms. Based on the matrix probability test of the ZigBee protocol randomness test algorithm, test sequence division, and test organization, a scientific and reasonable evaluation method and its theoretical guarantee are proposed in this study. Through simulation experiments, it was verified that the randomness of the output sequence of the ZigBee protocol can be tested. The test methods and ideas in this study can be widely used to assess the safety of ZigBee technology and to provide a theoretical basis and guidelines of operation for wireless sensor networks and Internet of Things security tests in practice. This method is of theoretical and practical significance.

In the next step, we will focus on the security detection methods of wireless sensor networks such as Bluetooth and WIFI. For the test conclusions, we will further study the more complete and reasonable analysis standards and provide more theoretical support for the information security assessment of the Internet of Things.

## Figures and Tables

**Table 1:** Verifying the accuracy of the probability test matrix.

Matrix Probability Test			Matrix Rank Test
$P_{c}$	$P_{c}{}_{\left(\mathrm{obs}\right)}$	$P_{c}-P_{c}{}_{\left(\mathrm{obs}\right)}$	$P_{-value}$
0.0314	0.02	0.0114	0.4813
0.0632	0.081	0.0178	0.7419
0.0183	0.053	0.0283	0.0852
0.0733	0.039	0.0343	0.423
0.084	0.051	0.0330	0.328
0.0399	0.065	0.0251	0.539
0.0924	0.05	0.0424 > 0.0422	0.0062 < 0.01 (no pass)
0.0673	0.037	0.0303	0.235
0.0388	0.089	0.0502 > 0.0422	0.0027 < 0.01 (no pass)
0.0247	0.09	0.0653 > 0.0422	0.0083 < 0.01 (no pass)
0.0815	0.01	0.0715 > 0.0422	0.000773 < 0.01 (no pass)
0.0166	0.078	0.0614 > 0.0422	0.000158 < 0.01 (no pass)
